# Supplementary figures and images for: MicroRNA panel in serum reveals novel diagnostic biomarkers for prostate cancer
Source: PeerJ. 2021 May 19;9:e11441. doi: 10.7717/peerj.11441 (PMC8141284; doi:10.7717/peerj.11441)

**A**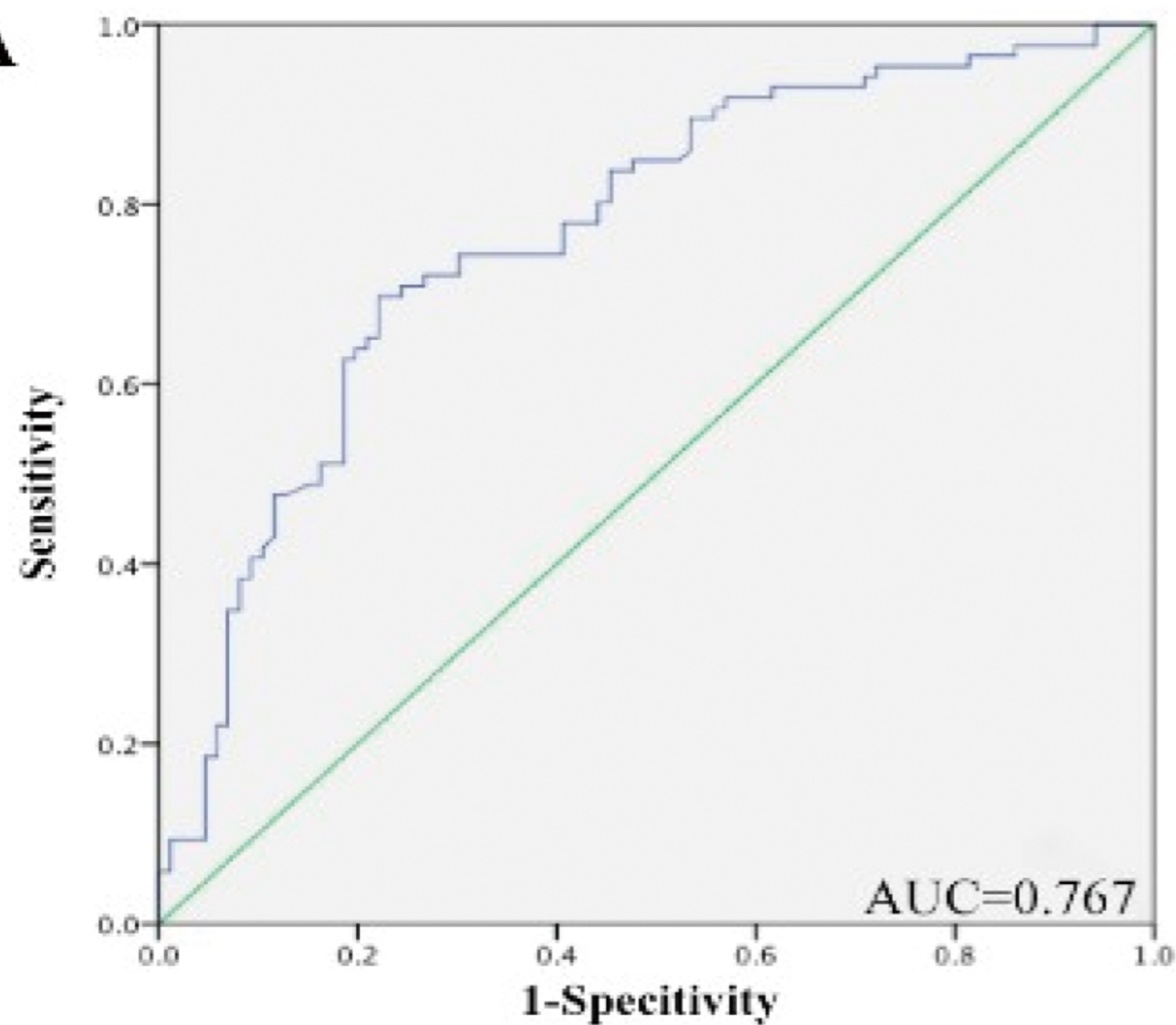**B**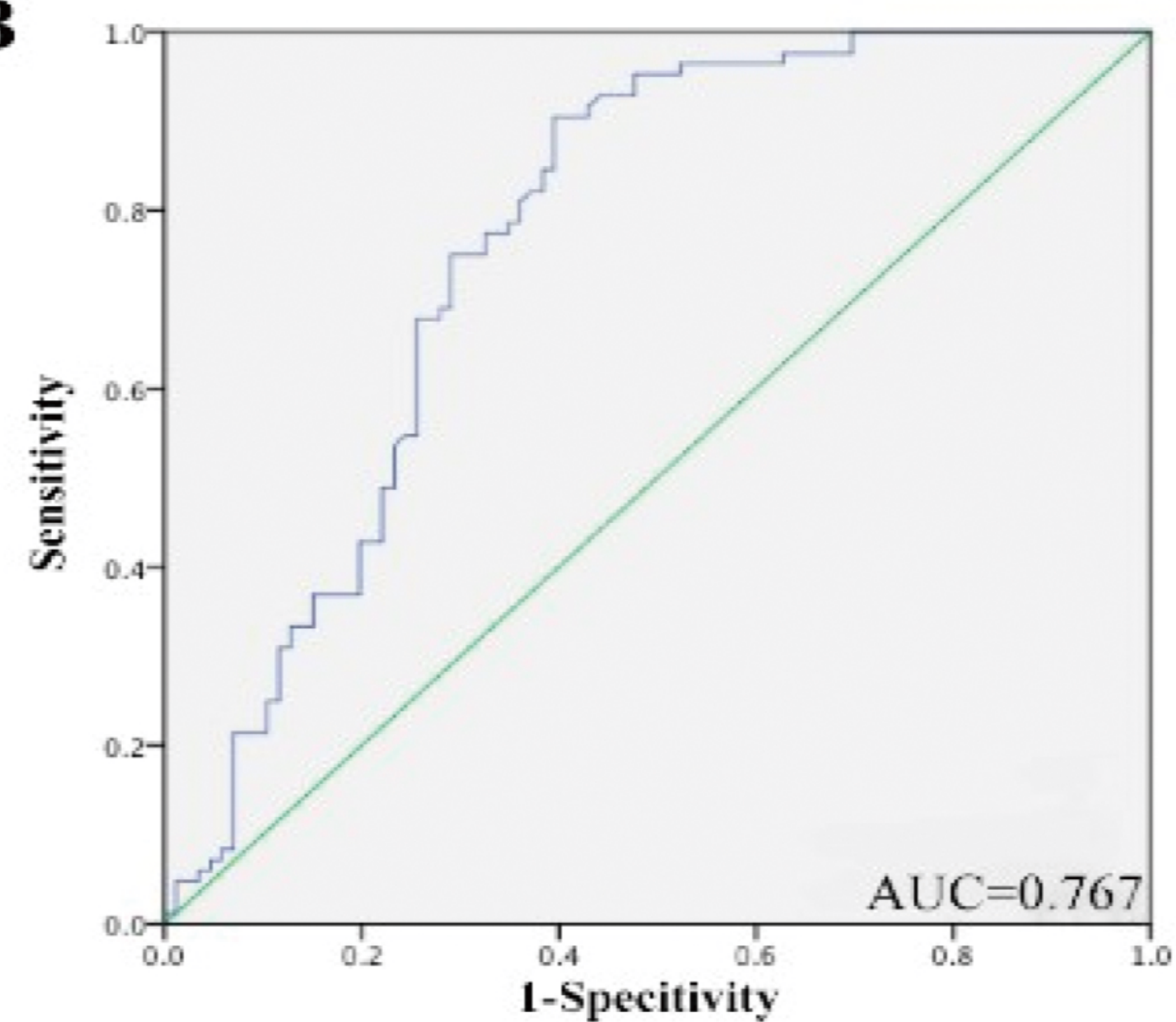**C**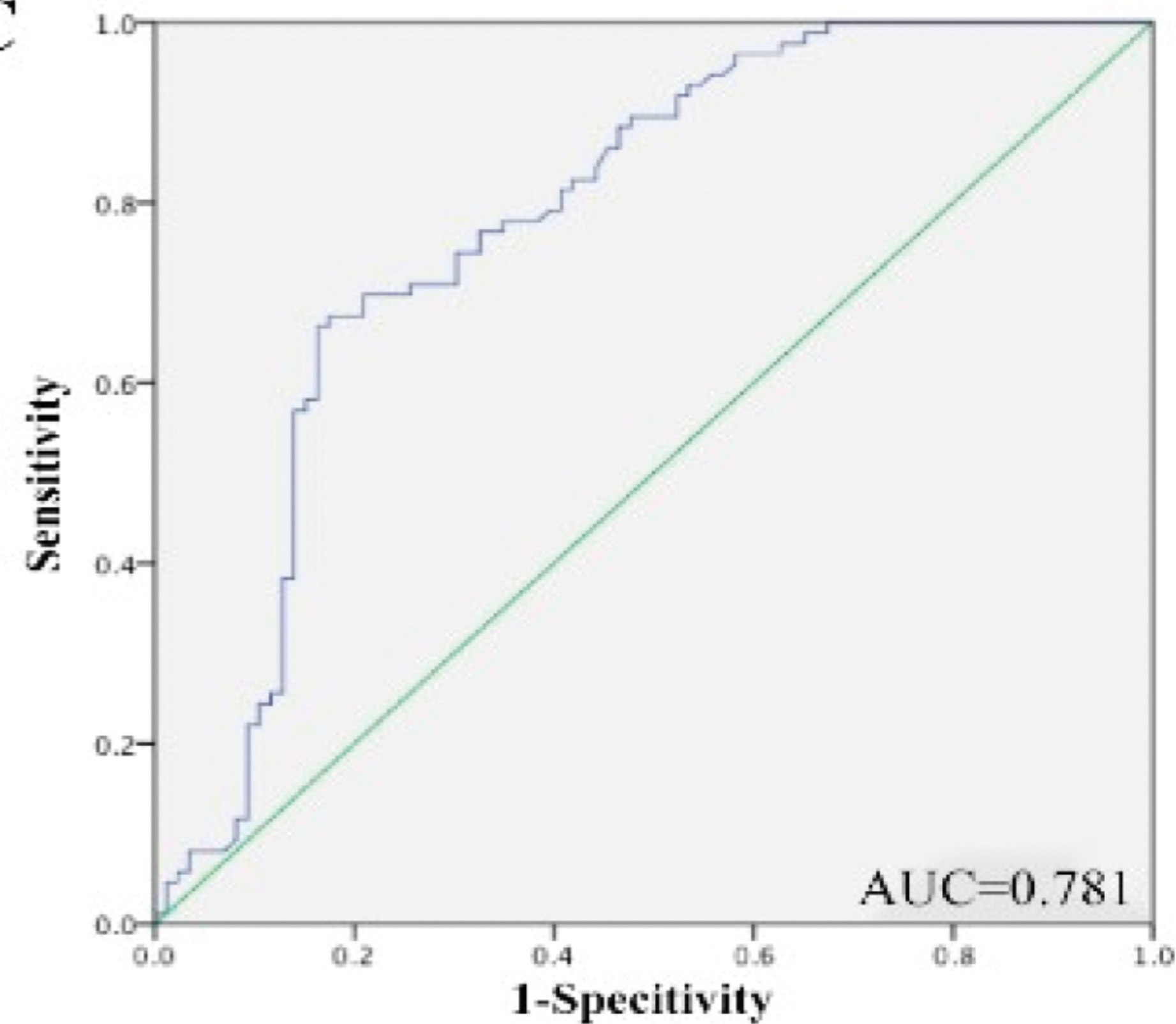

Supplement: Supplemental Information 1 — (A) miR-146a-5p. (B) miR-24-3p. (C) miR-93-5p. [file peerj-09-11441-s001.pdf]

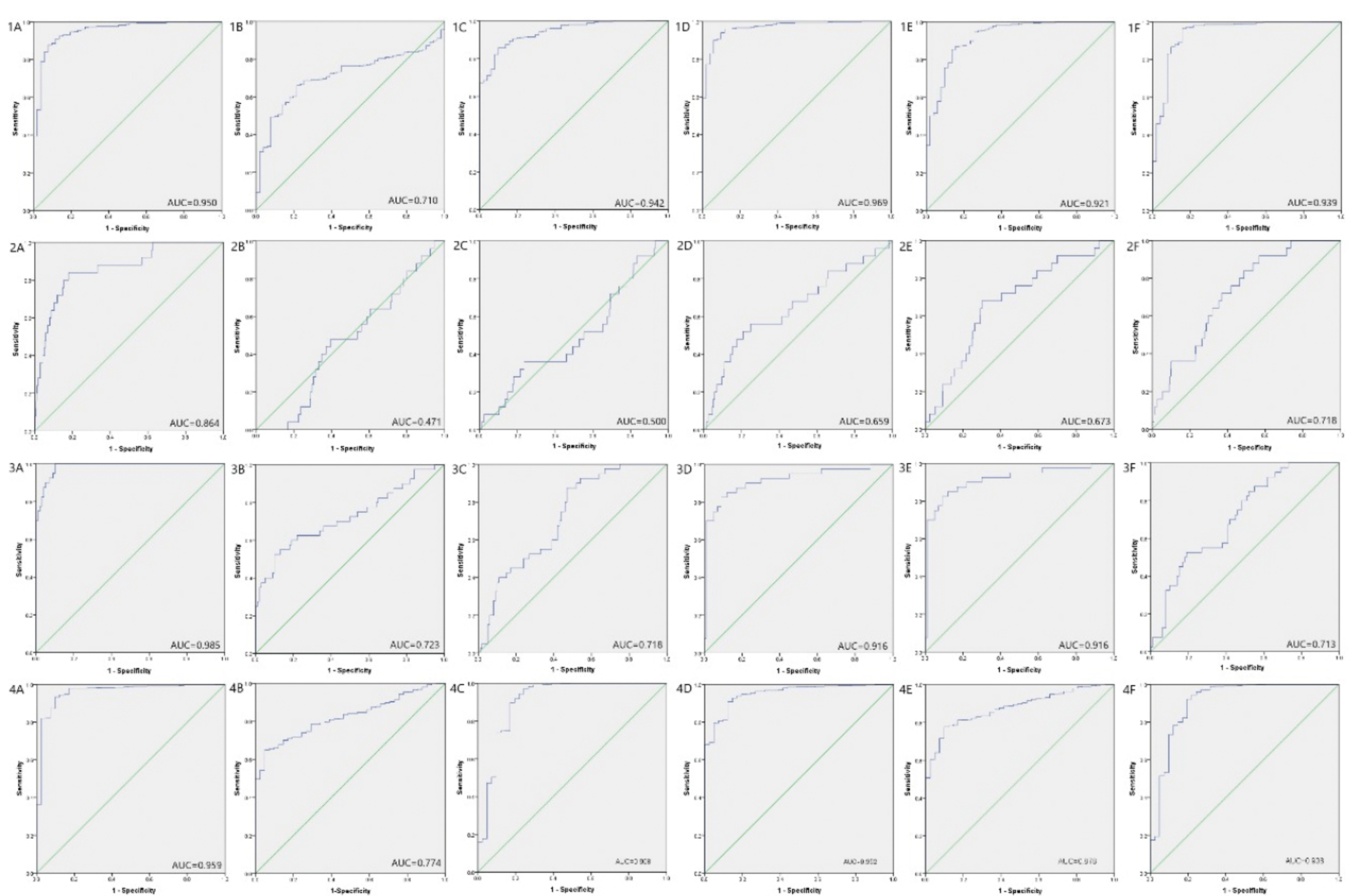

Supplement: Supplemental Information 2 — TCGA: (1A) Bryant R.J. et al.: AUC = 0.950, 95% CI [0.919–0.981], P < 0.001, Sensitivity = 87.8%, Specificity = 92.2%. (1B) Chen Z.H. et al.: AUC = 0.710, 95% CI [0.655–0.765], P < 0.001, Sensitivity = 66.1%, Specificity =78.4%. (1C) Yaman Agaoglu F. et al.: AUC = 0.942, 95% CI [0.916–0.967], P < 0.001, Sensitivity = 85.7%, Specificity =90.2%. (1D) Moltzahn F. et al.: AUC = 0.969, 95% CI [0.949–0.989], P < 0.001, Sensitivity = 96.0%, Specificity = 88.2%. (1E) Paunescu I.A. et al.: AUC = 0.921, 95% CI [0.880–0.963], P < 0.001, Sensitivity = 85.3%, Specificity = 86.3%. (1F) Porzycki P. et al.: AUC = 0.939, 95% CI [0.897–0.981], P < 0.001, Sensitivity = 96.6%, Specificity = 84.3%. GSE113740: (2A) Bryant R.J. et al.: AUC = 0.864, 95% CI [0.789–0.938], P < 0.001, Sensitivity = 84.0%, Specificity = 82.0%. (2B) Chen Z.H. et al.: AUC = 0.659, 95% CI [0.540–0.778], P = 0.007, Sensitivity = 56.0%, Specificity = 75.3%. (2C) Yaman Agaoglu F. et al.: AUC = 0.500, 95% CI [0.382–0.618], P = 0.999, Sensitivity = 36.0%, Specificity = 76.5%. (2D) Moltzahn F. et al.: AUC = 0.718, 95% CI [0.633–0.775], P < 0.001, Sensitivity = 84.0%, Specificity = 51.3%. (2E) Paunescu I.A. et al.: AUC = 0.673, 95% CI [0.570–0.775], P = 0.003, Sensitivity = 68.0%, Specificity = 70.0%. (2F) Porzycki P. et al.: AUC = 0.471, 95% CI [0.372–0.569], P = 0.616, Sensitivity = 48.0%, Specificity = 60.4%. GSE113486: (3A) Bryant R.J. et al.: AUC = 0.985, 95% CI [0.972–0.999], P < 0.001, Sensitivity = 100 .0%, Specificity = 90.0%. (3B) Chen Z.H. et al.: AUC = 0.723, 95% CI [0.620–0.827], P < 0.001, Sensitivity = 52.5%, Specificity = 90.0%. (3C) Yaman Agaoglu F. et al.: AUC = 0.718, 95% CI:0.631-805, P < 0.001, Sensitivity = 87.5%, Specificity = 53.0%. (3D) Moltzahn F. et al.: AUC = 0.916, 95% CI [0.856–0.976], P < 0.001, Sensitivity = 82.5%, Specificity = 91.0%. (3E) Paunescu I.A. et al.: AUC = 0.853, 95% CI:0.776-930, P < 0.001, Sensitivity = 80.0%, Specificity = 80.0%. (3F) Porzycki P. et al.: AUC = 0.713, 95% CI [file peerj-09-11441-s002.pdf]

Relative expression

miR-146a-5p

P=0.613

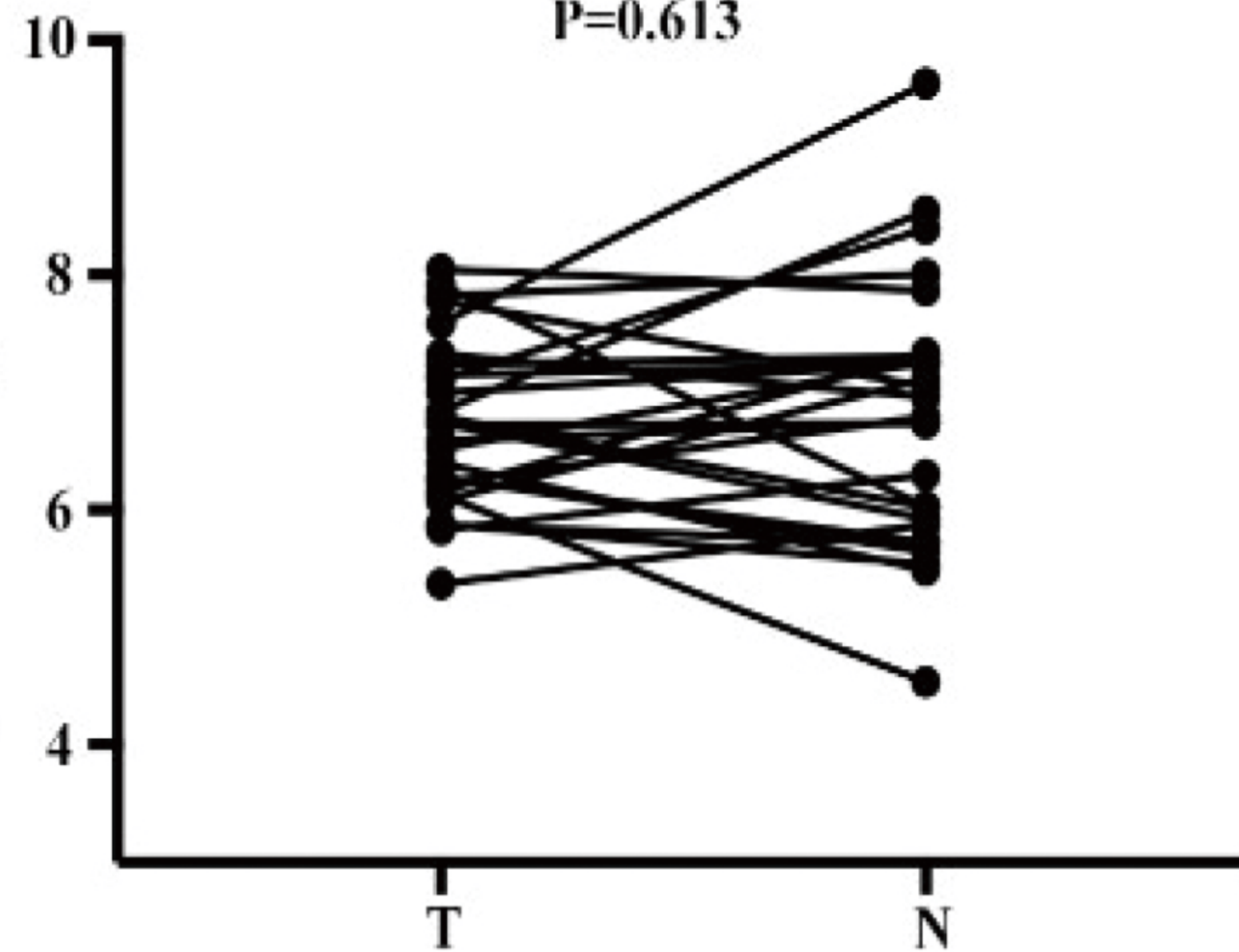

Relative expression

miR-24-3p

P=0.884

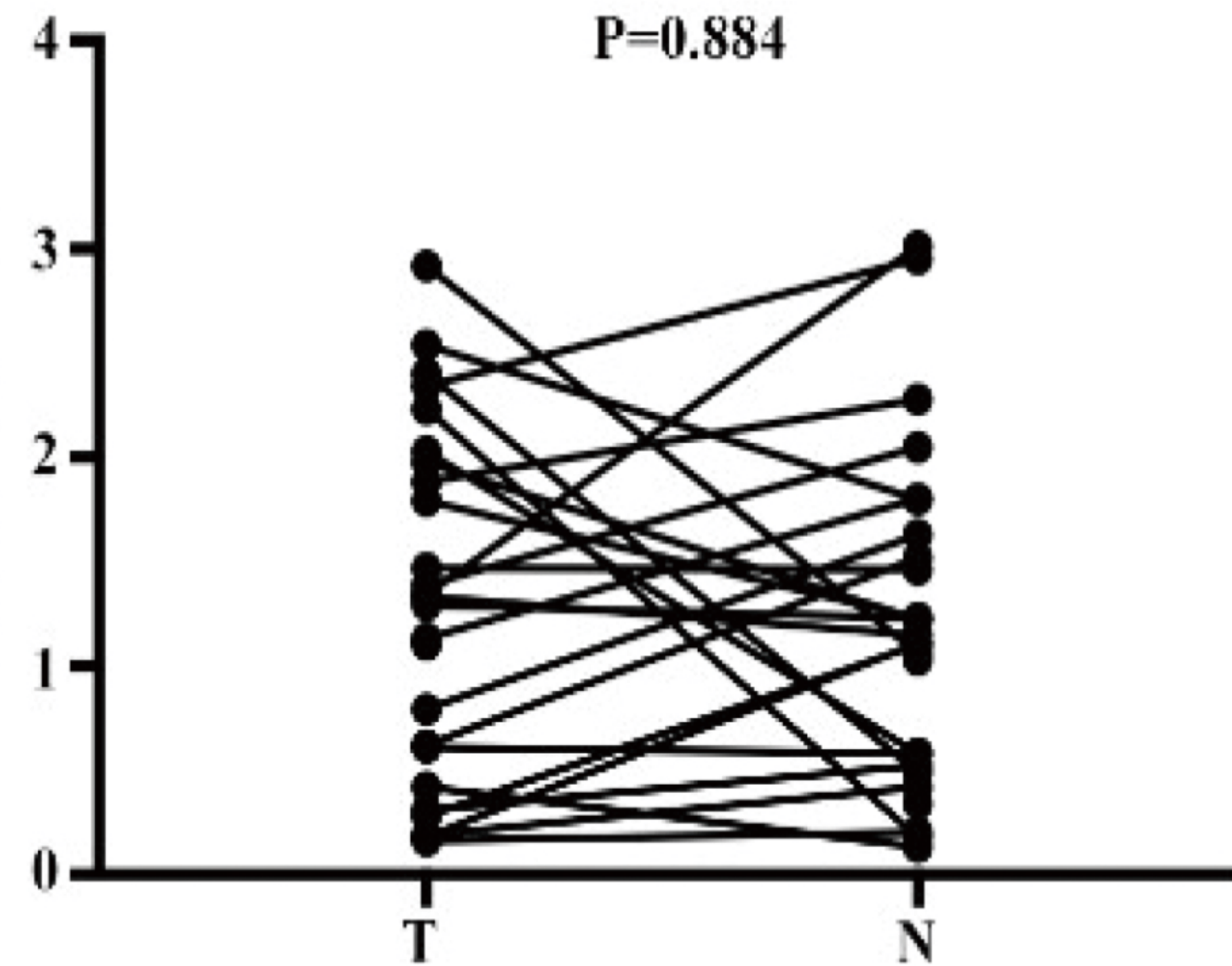

Relative expression

miR-93-5p

P=0.371

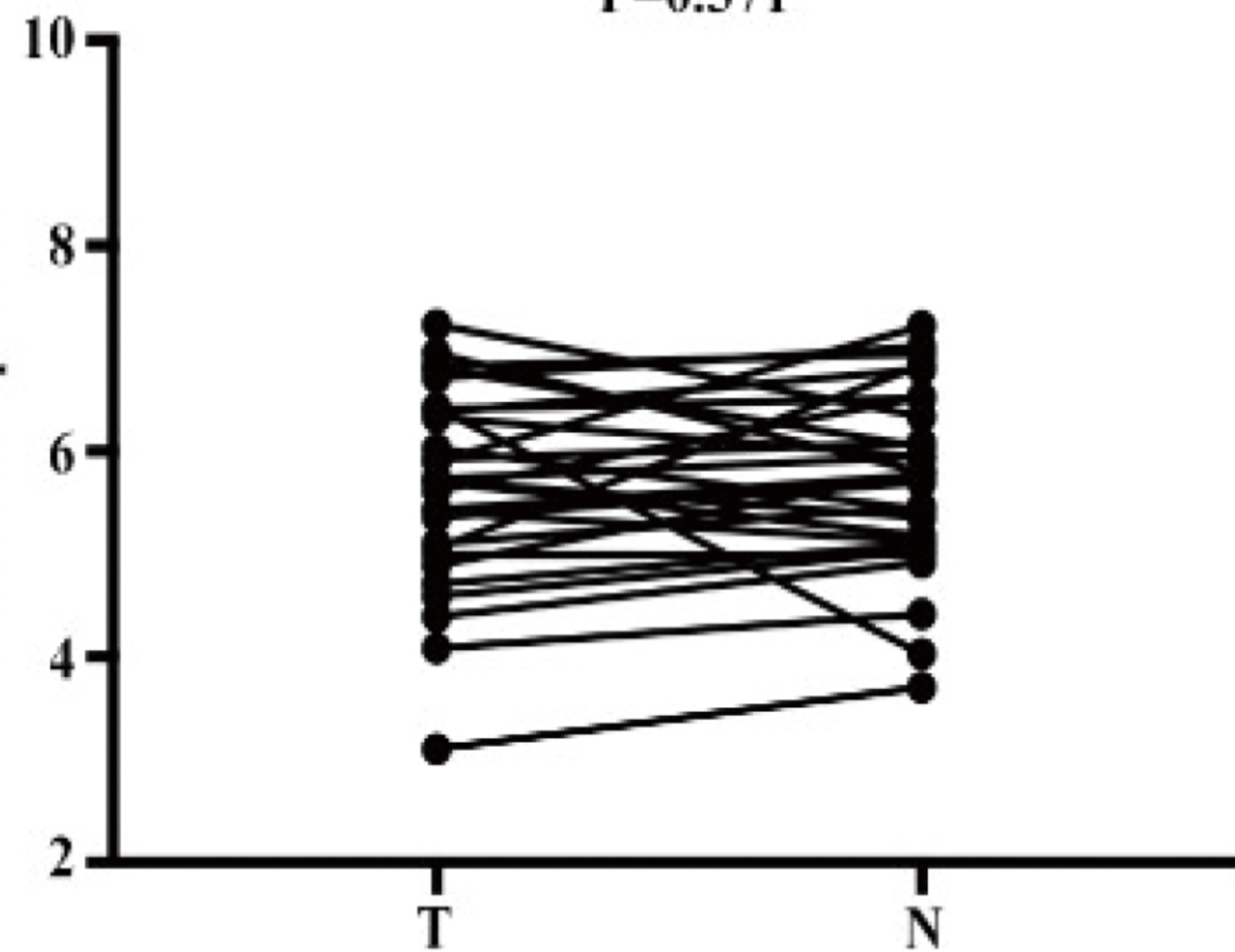

Supplement: Supplemental Information 3 — N, normal control; T, tumor. [file peerj-09-11441-s003.pdf]

A

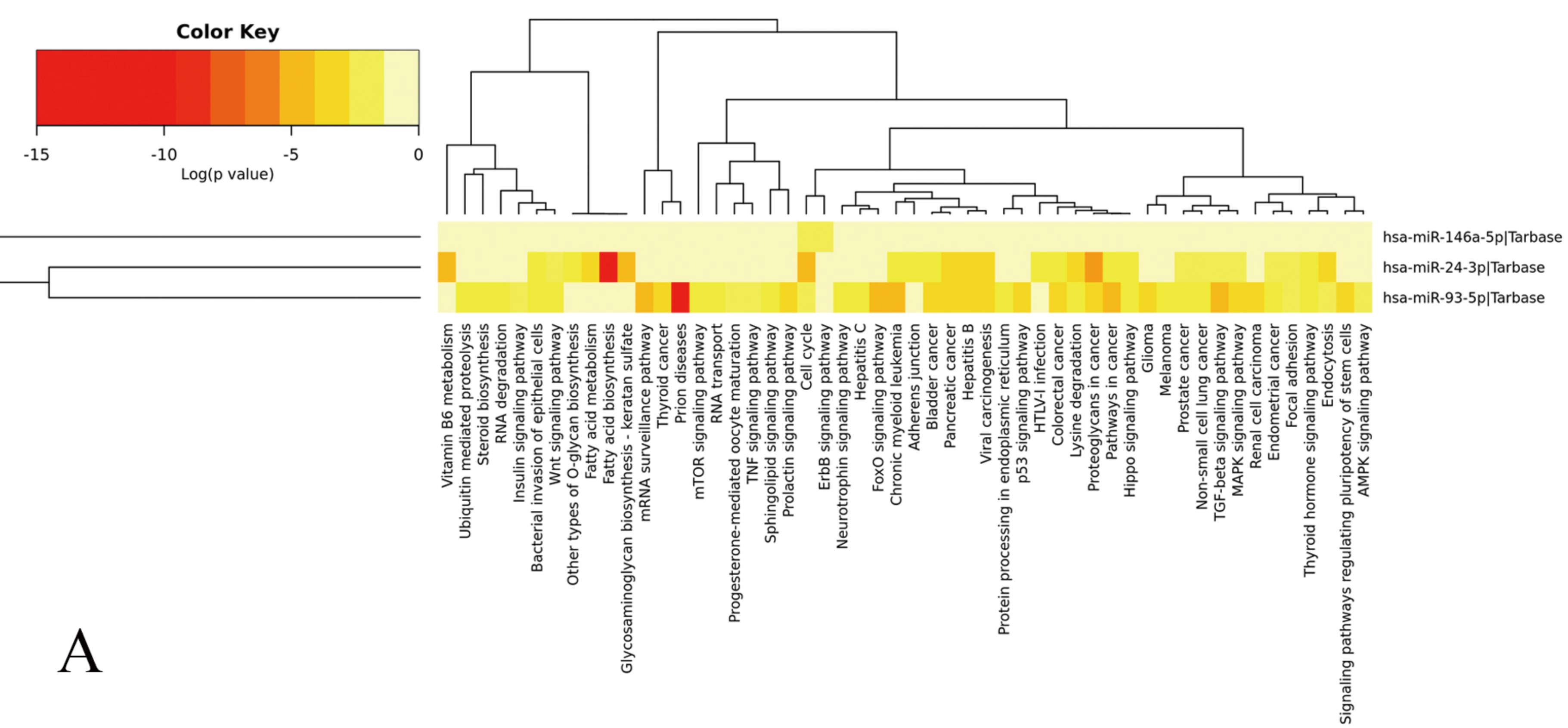

B

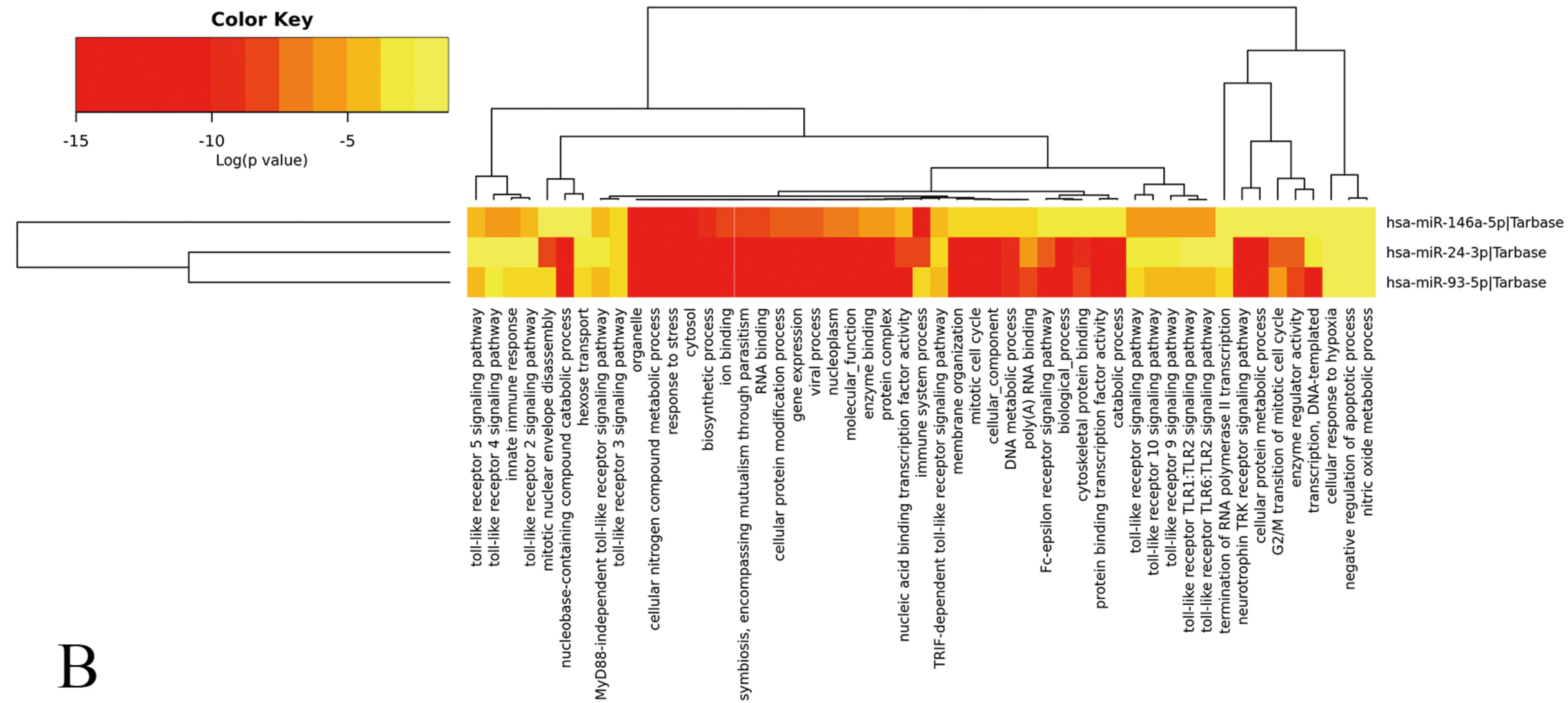

Supplement: Supplemental Information 4 — (A) KEGG, Kyoto Encyclopedia of Genes and Genomes. (B) GO, Gene Ontology. [file peerj-09-11441-s004.pdf]
