# Supplementary material for: MicroRNA panel in serum reveals novel diagnostic biomarkers for prostate cancer
Source: PeerJ. 2021 May 19;9:e11441. doi: 10.7717/peerj.11441 (PMC8141284; doi:10.7717/peerj.11441)
Supplement: Supplemental Information 6 — PC, prostate cancer; OS, overall survival; HR, hazard ratio; CI, confidence interval; T, tumor topography; N: lymph node. [file peerj-09-11441-s006.docx]

| **Variables** | **Univariate analysis** | | |
| --- | --- | --- | --- |
|  | **HR (95%CI)** | **P value** |  |
| Age (≥70 VS. ＜70) | 5.976(1.136,31.448) | 0.035 |  |
| T (3+4 VS. 1+2) | 3.283(0.582,18.511) | 0.178 |  |
| N (1 VS. 0) | 4.629(0.926,23.133) | 0.062 |  |
| miR-146a-5p (≥median VS. <median) | 0.504(0.126,2.021) | 0.334 |  |
| miR-24-3p (≥median VS. <median) | 0.849(0.187,3.868) | 0.833 |  |
| miR-93-5p (≥median VS. <median) | 1.241(0.292, 5.274) | 0.769 |  |
